# Improved Draft Genome Sequence of Probiotic Strain *Lactobacillus gasseri* K7

**DOI:** 10.1128/genomeA.00725-14

**Published:** 2014-07-24

**Authors:** Primož Treven, Aljoša Trmčić, Bojana Bogovič Matijašić, Irena Rogelj

**Affiliations:** University of Ljubljana, Biotechnical Faculty, Institute of Dairy Science and Probiotics, Domžale, Slovenia

## Abstract

*Lactobacillus gasseri* K7 is an isolate from infant feces and has *in vitro* and *in vivo* established probiotic properties. Here, we report the improved version of the draft genome sequence, which comprises 8 scaffolds (13 contigs), a total length of 1.99 Mb, and 1,841 predicted protein-coding sequences.

## GENOME ANNOUNCEMENT

*Lactobacillus gasseri* is an autochthonous microorganism that colonizes the gastrointestinal tract, oral cavity, and vagina in humans and animals. Strains of *L. gasseri* species are frequently declared to be probiotics (1). *L. gasseri* K7 is an isolate from infant feces and has established probiotic properties *in vitro* and *in vivo* (2–4). The gassericins K7 A (GenBank accession no. EF392861) and K7 B (GenBank accession no. AY307382) produced by this strain are among the first characterized bacteriocins of human-derived probiotic bacteria (5), which are particularly interesting due to a wide range of activities against Gram-positive bacteria, including *Clostridium difficile* and *Clostridium perfringens* (6, 7).

The *L. gasseri* K7 genome was sequenced using 454 Titanium GS FLX+ pyrosequencing (Roche, Branford, CT). The obtained reads were assembled using Newbler version 2.6. The resulting 51 contigs (maximum length, 207,105 bp; minimum length, 101 bp; *N*_50_ contig size, 86,593; mean coverage, 29×) were ordered against the complete genome sequence of *L. gasseri* ATCC 33323 using ABACAS (8). Gap closure was performed by Sanger sequencing of gap-closing PCR products obtained with primers generated with the Primer3 software (9). Gap closure resulted in a final 13 contigs, and the relationships against the reference genome of *L. gasseri* ATCC 33323 were maintained in the GenBank submission by the inclusion of an A Golden Path (AGP) file. Annotation and gene prediction were performed by the NCBI Prokaryotic Genomes Automatic Annotation Pipeline (PGAAP) (10) and the IMG-ER platform (11), which was also used to compare the genome of the *L. gasseri* K7 strain with other genomes. Before final submission, genome annotation was manually curated to correct the nomenclature of bacteriocins and insertion sequences. Artemis (12) and the IMG-ER platform were used for manual curation.

The submitted genome sequence of *L. gasseri* K7 comprises 8 scaffolds (13 contigs), with a total length of 1993,970 bp, 34.81% G+C content, and 1,841 predicted protein-coding sequences (CDSs). It includes 10 rRNA gene operons and 55 predicted tRNA genes. Compared to *L. gasseri* ATCC 33323, part (278,000 bp) of the *L. gasseri* K7 genome sequence is inverted. The results of a BLAST search against a plasmid-specific database available on the PATRIC website (13) indicate that contig005 is part of the plasmid sequence. PHAST analysis (14) revealed one complete (KC5α), one questionable (ϕADH), and two incomplete prophage regions. One incomplete and 19 complete repeats of insertion sequence ISLga*1* (IS*30* family) were identified by IS Finder (15) and a BLAST search. One clustered regularly interspaced short palindromic repeat (CRISPR) element (37 bp) with 14 spacers was detected using CRISPRFinder (16). Besides the previously described bacteriocins gassericin K7 A and K7 B (5), analysis with BAGEL3 (17) revealed two additional putative coding genes for bacteriocin helveticin-J and for gassericin A-like bacteriocin (class IIc). *L. gasseri* K7 has 103 genes with no homologs in other publicly available *L. gasseri* genomes. Among the unique genes, we found 4 putative amino acid ABC transporters, 3 different putative glucosidases, a putative arabinose transporter, and 4 different putative glycosyltransferases, which suggests that the strain is adapted to specific sugar- and amino acid-rich environments, such as the infant intestinal tract.

### Nucleotide sequence accession numbers.

This whole-genome shotgun project has been deposited at DDBJ/EMBL/GenBank under the accession no. ASRG00000000. The version described in this paper is version ASRG02000000.
